# Pollen and anther morphological variation in rye was shaped by domestication

**DOI:** 10.1186/s12870-025-06416-x

**Published:** 2025-03-27

**Authors:** Christina Waesch, Yixuan Gao, Natalie Koch, Noah Gaede, Thomas Hornick, Christian Dusny, Jörg Fuchs, Andreas Börner, Axel Himmelbach, Martin Mascher, Klaus Pillen, Susanne Dunker, Steven Dreissig

**Affiliations:** 1 Institute of Agricultural and Nutritional Sciences, Martin-Luther-University Halle- Wittenberg, Halle (Saale), Germany; 2 Helmholtz-Centre for Environmental Research – UFZ Leipzig, Leipzig, Germany; 3 German Centre for Integrative Biodiversity Research (iDiv) Halle-Jena-Leipzig, Leipzig, Germany; 4 Leibniz Institute of Plant Genetics and Crop Plant Research (IPK), Seeland, OT, Gatersleben Germany

**Keywords:** Domestication, Pollen morphology, Anther morphology, Poaceae, Within-species diversity, Multispectral imaging flow cytometry

## Abstract

**Background:**

In plants and animals, pollen or sperm morphology differ greatly between species. Across plant species, pollen morphological diversity is broadly linked to different pollination systems. However, the extent of within-species diversity is less well understood. To address this question, we explored pollen and anther diversity in rye (*Secale cereale* L.), a wind-pollinating grass species.

**Results:**

We analysed 339 domesticated, feral and wild rye individuals of 64 diverse accessions. Population structure analysis revealed a differentiation gradient from wild to domesticated rye. We found pronounced within-species diversity of pollen and anther morphology. Genome-wide association scans uncovered a polygenic architecture of pollen and anther traits, with medium to high heritability and mostly small-effect loci. A subset of these loci overlapped with previously identified domestication loci, for which the underlying traits were unknown. A *P*_*ST*_-*F*_*ST*_ analysis suggests that pollen and anther traits were under selection throughout rye domestication. Population genomic analyses revealed signatures of selection at 37% of all identified loci.

**Conclusion:**

Our work shows that selection for larger pollen grains and longer anthers occurred throughout rye domestication. The present study extends our knowledge of the genetic architecture underlying within-species pollen and anther morphological diversity, and further unravels domestication traits in rye.

**Supplementary Information:**

The online version contains supplementary material available at 10.1186/s12870-025-06416-x.

## Background

Cross-pollination provides an important mechanism for creating novel allelic combinations in a population, which may facilitate adaptation to changing environments. The development of viable pollen is a crucial part of the life cycle of plants ensuring sexual reproduction and seed set. The overall morphology of pollen grains is related to their respective pollination mechanism [1, 2]. Insect-pollinated species exhibit pronounced sculpting of the outer pollen cell wall (exine), while wind-pollinated species display smooth exine structures devoid of pollenkitt, a sticky pollen coat material, thus minimizing the risk of clumping [3–5]. Wind-pollinating plants appear to allocate more resources in the production of large amounts of pollen, generally showing a higher number of pollen produced per ovule (pollen-ovule ratio), which increases the chances of successful fertilization [6]. A limiting factor for long-distance wind dispersal is pollen size. Smaller pollen have a lower mass, which reduces their settling velocity and increases dispersal distance from the parental plant [7, 8]. Consequently, it is reasonable that wind-pollinating plants produce smaller pollen in a defined range of 20–60 μm, with reduced viability, for example lasting only a few hours in the Poaceae family. Zoophilous pollen, on the other hand, is ranging from 5 to 200 μm and stay viable for several days to months [6, 8–11]. Pollen size also correlates with the amount of stored nutrients, meaning a decreased nutrient content due to smaller size [12–14]. Lower nutrient levels might affect pollen germination by decelerating pollen tube growth, but would also lower the risk of animal attraction and consumption [15].

Rye (*Secale cereale* L.) is a self-incompatible, wind-pollinating cereal grass belonging to the family of Poaceae and the Triticeae tribe along with wheat (*Triticum aestivum*) and barley (*Hordeum vulgare*). Unlike its relatives, rye was domesticated outside the Fertile Crescent after exploiting Vavilovian mimicry and thriving as a weed within wheat and barley fields [16–19]. The genus *Secale* is comprised of three partially interfertile species: *S. cereale* (annual), *S. sylvestre* (annual) and *S. strictum* (perennial). The latter two species are found only in the wild [18, 20–22]. *S. cereale*, by contrast, includes its putative wild progenitor *S. cereale* subsp. *vavilovii*, domesticated rye *S. cereale* subsp. *cereale* and feral or weedy subspecies with strong gene flow between the groups owing to cross-pollinating and the self-incompatible nature of rye [18, 23].

Many crop domestication studies identified single large-effect loci associated with domestication traits like non-shattering seeds, increased seed size, and reduced seed dormancy in crops like wheat, maize, and rice. More recently, higher genomic resolution analysis supports a polygenic architecture of these domestication phenotypes [24]. A recent study in *Secale* identified signatures of selection at loci with candidate genes linked to plant fertility and reproduction [25]. The floral characteristics of rye were extensively investigated in the past. These studies demonstrated that rye typically has long anthers (8–9.5 mm), accompanied by a high pollen count per anther (22,000–42,000), and its pollen exhibits prolonged viability compared to self-pollinating wheat [10, 26–28]. The genetic architecture underlying these traits remains elusive, but modulating out-crossing capacities in crops presents an opportunity to improve hybrid breeding programs.

Here, our aim was to uncover the genetic architecture underlying quantitative variations in pollen and anther morphology within an outbreeding and wind-pollinating grass species (*Secale cereale* L.). We hypothesized that within-species morphological variation was driven by allelic variation, and that selection of such alleles may have occurred during domestication. This was based on the observation that floral traits were shown to differ between and within species [1]. However, a comprehensive survey of population-level within-species variation, and especially of the underlying genetic architecture, was not yet conducted. For this purpose, we analysed 339 rye individuals derived from a diverse set of 64 rye accessions of different geographic origin and domestication status [18, 23]. We analysed population structure based on reduced representation sequencing data (genotyping-by-sequencing, GBS), characterized pollen and anther morphological traits using high-throughput multispectral imaging flow cytometry (MIFC) as well as conventional microscopy, performed genome-wide association scans to uncover the underlying genetic architecture, and analysed whether pollen and anther traits were selected for during the domestication of rye. In doing so, we discovered heritable variation in pollen and anther traits, with a mostly polygenic architecture. A *P*_*ST*_-*F*_*ST*_ analysis revealed that pollen and anther traits were selected for throughout the domestication of rye, with significant signatures of selection at a subset of loci.

## Methods

### Plant material, field trial and sample collection

In this study we used a diverse set of 64 domesticated, feral, and wild genebank accessions of *S. cereale* L., which were derived from a previously described diversity collection [18, 23]. Per accession, we analysed one to six individuals, resulting in a total of 339 individual genotypes (Supplementary Table 1). The rye accessions ranged from domesticated (234) including old cultivars (146) and landraces (88), to feral (84) and wild rye including *S. c*. subsp. *vavilovii* (17) and *S. strictum* subsp. *anatolicum* (4). In this context feral refers to rye which was once domesticated but became weedy again, whereas wild designates either the wild progenitor *S. c*. subsp. *vavilovii* or wild perennial *Secale strictum*. Passport information of these accessions (taxonomic status, country of origin, collection site) is available at the genebank information system of the German Federal ex situ Genebank at IPK Gatersleben and in Supplementary Table 1 (GBIS; https://gbis.ipk-gatersleben.de/GBIS_I [29]). Plants were cultivated on the Experimental Field Station of the Martin-Luther-University in Halle (Saale), central Germany (51°29′52.7′′N, 11°59′31.3′′E), during the growing season 2021/2022. Per accession 10 grains were sown in double rows in a plot size of 30 cm x 100 cm and a row and plot space of 10 cm and 30 cm, respectively. The plots were not treated with additional fertilizers or growth regulators, only once with herbicide Biathlon^®^4D (70 g/ha) in combination with Dash^®^E.C. (1 /ha). The majority of our plant material comprised winter rye (265), which was sown with perennial *S. strictum* in autumn 2021, whereas the spring types (70) were sown in spring 2022. It should be noted that our experiments were based on single plants. Since rye is a self-incompatible, cross-pollinating species, individual plants represent a distinct genotype, hindering biological replications of genotypes. In our work, we define a flower as a rye spikelet composed of two florets, each floret containing three anthers. We collected four to six flowers per individual of the main tiller from the centre of the spike prior to anther dehiscence with yellow anthers still remaining inside the flower. Flower samples were transferred into a 1.5 ml Eppendorf tube containing 1 ml of fixative (3:1 ethanol (99%): glacial acetic acid (99%)) and were stored at 4 °C.

### Ploidy level Estimation

Ploidy level was determined according to [30] for each genotype of 51 accessions (Supplementary Fig. 1) by taking leaf samples at the seedling stage. Initially, the ploidy level of the first accession was measured together with leaf nuclei of diploid *Hordeum vulgare* L. as a reference. Ploidy levels of the remaining genotypes were estimated based on a comparative analysis of proportion of heterozygosity using the individuals of known ploidy as reference (Supplementary Fig. 2) [31]. Proportion of heterozygosity was calculated using VCFtools based on genotyping-by-sequencing (GBS) data (described below) [32].

### Genotyping by sequencing (GBS)

Leaf samples were collected from each individual at seedling stage and DNA was isolated using the DNeasy Plant Mini Kit (Qiagen, Hilden, Germany). Genotyping-by-sequencing (GBS) was done as described in [18], and read alignment and variant calling was performed against the Lo7 reference genome assembly [21] as described in Schreiber et al. (2022). Raw sequence data are available at the European Nucleotide Archive under project number PRJEB75239. The resulting single nucleotide polymorphism (SNP) matrix in Variant Call Format was filtered for minimum read depth per single nucleotide polymorphism (SNP) of 4 (--minDP 4), maximum missing data of 10% (--max-missing 0.9), minor allele frequency of 0.05 (--maf 0.05), to contain only bi-allelic sites (--min-alleles 2 --max-alleles 2), and no indels (--remove-indels) using VCFtools [32]. The final SNP matrix contained 56,713 sites, with an average SNP density of 8.5 SNPs/Mb and an average coverage of 19.1 reads per site. In order to analyse population structure, we performed a principal component analysis (PCA) based on a genetic covariance matrix using the snpgdsPCA() function in the SNPRelate package in R [33].

### Anther and pollen phenotyping

The following traits of anther and pollen morphology were assessed: anther length (AL in mm), pollen length (PL in µm), pollen area (PA in µm²), pollen elongatedness, the ratio of pollen length and width, (PE in arbitrary units (a.u.)), pollen mass (PM in ng), green (PGFI in a.u.), orange (POFI in a.u.), yellow (PYFI in a.u.) and red fluorescence intensity of pollen (PRFI in a.u.). We refer to pollen length as a measure of the longest axis of the pollen.

### Measurement of anther length

Anthers (total of 6 anthers out of 1 flower per individual) were extracted from a single fixed flower sample using tweezers, and arranged and imaged using a stereo microscope (50x magnification) equipped with a camera (Stemi 508, Zeiss; AxioCam ERc 5s, Zeiss). Anther length was measured using image analysis software ImageJ (http://imagej.nih.gov/ij/index.html) and the median per individual was calculated based on six anthers.

### Pollen sample Preparation

Pollen morphology was analysed using multispectral imaging flow cytometry (MIFC) following a protocol modified from [34]. Per individual, a single anther was extracted from a fixed spikelet and gently disrupted in a new 1.5 ml Eppendorf tube containing 3:1 ethanol (99%):glacial acetic acid (99%) using a pestle. The pollen suspension was vortexed for 30 s and sonicated for 5 min at room temperature in an ultrasonic bath (Transsonic 460/H, Roth Karlsruhe, Germany). The sample was centrifuged for 2 min at 4000 *g* (Mikro 200R, Hettich GmbH, Germany), the supernatant was carefully removed, and the pollen pellet was resuspended in 1 ml 1x Dulbecco’s phosphate buffered saline (without calcium, without magnesium) (D-PBS) (Biowest, Nuaillé, France). Cellular debris was removed from the pollen suspension by filtering and transferring it into a new 1.5 ml Eppendorf tube using a 100-µm mesh filter (CellTrics, Sysmex Partec GmbH, Germany). Subsequently, the samples were centrifuged for 2 min at 4000 *g* and the supernatant volume was adjusted to 100 µl 1x D-PBS. Samples were stored at 4 ⁰C overnight or at -20 ⁰C for longer periods. Before measurement, pollen samples were vortexed, and a volume of 30 µl of the pollen suspension was dispensed into an individual well of a 96-well plate. Subsequently, the plate was introduced into the multispectral imaging flow cytometer ImageStream^®^ X MK II (Amnis part of Luminex, Austin, Texas, USA) autosampler. The ImageStream^®^ X MK II system is equipped with three lasers (488 nm laser with 5 mW intensity, 561 nm laser with 20 mW intensity (incl. neutral density filter 1.0), and 785 nm laser with 0.1 mW intensity) and two CCD cameras and can record at once 12 images for each particle, including brightfield, fluorescence and scatter images The measurement of each sample was terminated when either 5000 pollen particles were imaged or if less particles were measured after a period of 6 min. To avoid sample loss caused by evaporation, only half of a 96 well plate was used in each run.

### Pollen image gating and morphological trait extraction

Images were captured at 20x object magnification with the instrument-specific INSPIRE Software (v.200.1.620.1). For each object passing through the cytometer, 12 distinct images (two brightfield, nine fluorescent, and one scatter image(s)) were captured. Most important for our analysis were images captured by the first camera (488 nm laser excitation) of channel 1 (brightfield image) and the fluorescence images of channel 2 (528/65 nm BP filter), channel 3 (577/35 nm BP filter), channel 4 (610/30 nm BP filter) and channel 5 (702/85 nm BP filter). The IDEAS software (v.6.2.187.0) was used to extract high-quality, single pollen images of fertile pollen based on a step-wise gating approach (Supplementary Fig. 3). For all brightfield image features the “AdaptiveErode (M01, Ch01, 91)” mask was applied. First, images were filtered based on pollen diameter (channel 1) in a range of 20–100 μm. Then, this fraction was filtered based on brightfield intensity (-1.2*10^6^ – -3*10^5^ a.u.) and median pixel intensity (-500 – -100) for channel 1 which ensured the exclusion of aborted pollen images. In order to remove stacked pollen images, filtering based on the brightfield features symmetry 3 (0–3) and symmetry 4 (0–3) of channel 1 was done. Finally, low-resolution images were eliminated by filtering based on correlation mean (0.2–0.6) and gradient RMS (8–19) of channel 5. Eventually, the data information of the single, high-quality and fertile pollen fraction was converted into FCS file format and the data were analysed within the R (4.1.3) environment. Individuals featuring 50 or more pollen images were incorporated. Following this, outlier exceeding the 99% quantile and below the 1% quantile considering pollen length were removed from the data set. In our experiment pollen length is defined as a measure of the longest axis of the pollen. This allowed us to measure an average of 600 fertile pollen grains per individual. Finally, median of all pollen morphology traits was calculated per individual and used for further analysis.

### Pollen mass measurement

Pollen mass was determined using quantitative phase imaging (QPI) by measuring the phase shift of light passing through a pollen, allowing to determine the refractive index of the pollen, which is correlated with its mass [35, 36]. Pollen mass was measured in a subset of 27 individuals representing the highest and lowest values of pollen and anther length. Samples of pollen pellets were prepared as described above (see Pollen sample preparation) but stored in 3:1 ethanol (99%):glacial acetic acid (99%). Agarose pads were prepared by transferring 400 µl of 1.5% low-melt agarose on a circular cover slide and placing a second cover slide on top. After the agarose solidified, the top cover slide was removed with tweezers and 15 µl of pollen suspension was transferred centrally on the agarose pad. Brightfield microscopic images of more than 100 pollen per individual with 100x magnification were taken using an inverted microscope (Axiocam 503; Carl Zeiss Microscopy GmbH) equipped with the camera wavefront sensor c-mounted (SID-4-sC8 sCMOS, Phaesics, France). As a reference, an image of the agarose pad without any pollen was taken. The captured pollen images were analysed using SID4BIO-1031 software (v 2.4.4., Phaesics, France) by manually segmenting the borders of each pollen to assess the optical thickness and thereby its mass.

### Phenotypic data analysis

All statistical analyses were carried out using the R (4.1.3) environment. Pearson’s correlation coefficients were calculated with the rcorr() function within the Hmisc package. Group comparisons were executed by Mann-Whitney-U-Test with wilcox_test() applying Bonferroni correction to control for multiple testing within the rstatix package.

### Genome-wide association scans and SNP-based heritability

A total of 314 and 286 genotypes were used for genome wide association scans (GWAS) for anther and pollen morphology traits using 55,475 SNPs, respectively. Tetraploid individuals (R944 and R1090) and *S. strictum* accessions were excluded from the analysis. GWAS was conducted using the fixed and random model circulating probability unification (FarmCPU) method in GAPIT (version 3) [37], which iteratively uses a fixed effect model (y = s_i_ + S + e), where s_i_ is the testing marker and S a pseudo quantitative-trait-locus (QTL), and a random effect model (y = K + e), where K is the kinship according to [38]. The fit of the model is indicated by quantile-quantile (QQ) plots for the respective traits (Supplementary Fig. 4). Additionally, we performed a 1,000-fold repeated random subsampling approach by selecting 95% of the original data set randomly in each run. The detection rate for each marker was computed as the relative abundance of the SNP exceeding the significance threshold across 1,000 runs (Supplementary Table 2). Further on, we performed permutations of 1,000 GWAS runs by randomly assigning phenotypic information to genotypes (Supplementary Table 3).

Candidate genes were screened within a +/- 5 Mb window around the significant associated SNP, which was based on LD-decay observed in our population (Supplementary Fig. 5). Thereby LD was calculated for an inter-SNP distance of 1–20 Mb using --geno-r2 function in using VCFtools [32]. Genes closest to a significant SNP were considered candidate genes (Supplementary Table 4). SNP-based heritability was calculated according to the method of [39] using gcta64. Therefore, the filtered VCF file was transformed into a PLINK file based on which a genetic relationship matrix was estimated with --make-grm. Based on the genetic relationship matrix and phenotypic information, the SNP-based heritability was calculated using --grm function for each trait (Supplementary Table 5).

### Population genomic analysis

For population genomic analysis, the initial SNP matrix was filtered for minimum read depth per single nucleotide polymorphism (SNP) of 10 (--minDP 10), maximum read depth per site of 100 (--maxDP 100), maximum missing data of 30% (--max-missing 0.7), minor allele frequency of 0.01 (--maf 0.01), to contain only bi-allelic sites (--min-alleles 2 --max-alleles 2), and no indels (--remove-indels) using VCFtools [32]. The final SNP matrix for population genomic analysis contained 70,954 SNPs. Pairwise *F*_*ST*_ values, nucleotide diversity (*π*), and cross-population composite likelihood ratio (XP-CLR) scores were estimated in 1 Mb windows with an overlap of 250 Kb using VCFtools and xpclr [32, 40]. For this analysis, we selected a domesticated and a feral subpopulation based on population structure information provided by PCA, with each comprising 30 individuals (Supplementary Table 6). Estimates of *π* were used to calculate the diversity reduction index (DRI = *π*_*feral*_ / *π*_*domesticated*_). Only 1 Mb windows containing at least 1 SNP were included. *F*_*ST*_, DRI, or XP-CLR values above the genome-wide 95% percentile were considered significant. To test if QTL showed signatures of selection, we screened genomic windows of +/- 5 Mb surrounding a QTL for significant *F*_*ST*_, DRI, or XP-CLR values (Supplementary Table 7).

### *P*_*ST*_*-F*_*ST*_ analysis

*P*_*ST*_*-F*_*ST*_ comparisons were performed for genotypes of the two domesticated and feral subpopulations for which also phenotypic information of anther and pollen morphology was available (Supplementary Table 8). This analysis serves to investigate if the observed phenotypic differences between the two subpopulations was caused by genetic drift (*P*_*ST*_ = *F*_*ST*_) or selection (*P*_*ST*_ > *F*_*ST*_) [41]. *P*_*ST*_ is used as an approximate of *Q*_*ST*_, and defined as the proportion of phenotypic variance explained by the population without differentiation between the relative impacts of genetic and environmental variations [42, 43]. *P*_*ST*_ is calculated utilizing the following formula: $\:{P}_{ST}=\frac{(c/{h}^{2}){\sigma\:}_{b}^{2}}{(c/{h}^{2}){\sigma\:}_{b}^{2}+2{\sigma\:}_{w}^{2}}$, where $\:{\sigma\:}_{b}^{2}$ and $\:{\sigma\:}_{w}^{2}$ represent the between and within phenotypic variance of the populations, and c/h^2^ is denoted as the proportion of additive variance across populations (c) relative to the within-population heritability (h^2^). We computed *P*_*ST*_ values using the R package pstat v.1.2 [44]. Since we used a common garden experiment, we assumed c and h^2^ to be equal (csh=1) [42, 44]. We calculated *P*_*ST*_ using 95% confidence intervals (opt=”ci”) of 1,000 resampled bootstraps (boot=1000). Genome-wide *F*_*ST*_ was calculated for the subgroups using VCFtools with the function --weir-fst-pop [32].

### Pollen dispersal modelling

We estimated the pollen dispersal distance using a model-based approach. Pollen settling velocity (V_set_) in still air was estimated using the equation described in [45] with environmental constants derived from [46] and [46], with acceleration of gravity *g* (m s^− 2^), the density of air (⍴_*m*_ = 1.225 kg m^− 3^), the viscosity of air (*µ* = 1.78 × 10^− 5^ kg m^− 3^ s^− 3^), a pollen diameter (*d*) and a pollen density of ⍴_*p*_ = 0.988 g cm^− 3^ (1).

1 $$\:{V}_{set}=\:\frac{{gd}^{2}({\rho\:}_{p}-{\rho\:}_{m})}{18\mu\:}$$

Dispersal distance (*D*) was calculated a ballistic equation formulated by [47] using pollen settling velocity (*V*_*set*_), the pollen release height (*h*_*r*_) and the horizontal wind speed ($\:\stackrel{-}{u}$) of 3 m/s (2).

2 $$\:D=\:\frac{{h}_{r}\stackrel{-}{u}}{{V}_{set}}$$

## Results

### Detection of domestication gradient within population structure of *S. cereale*

**Fig. 1:**
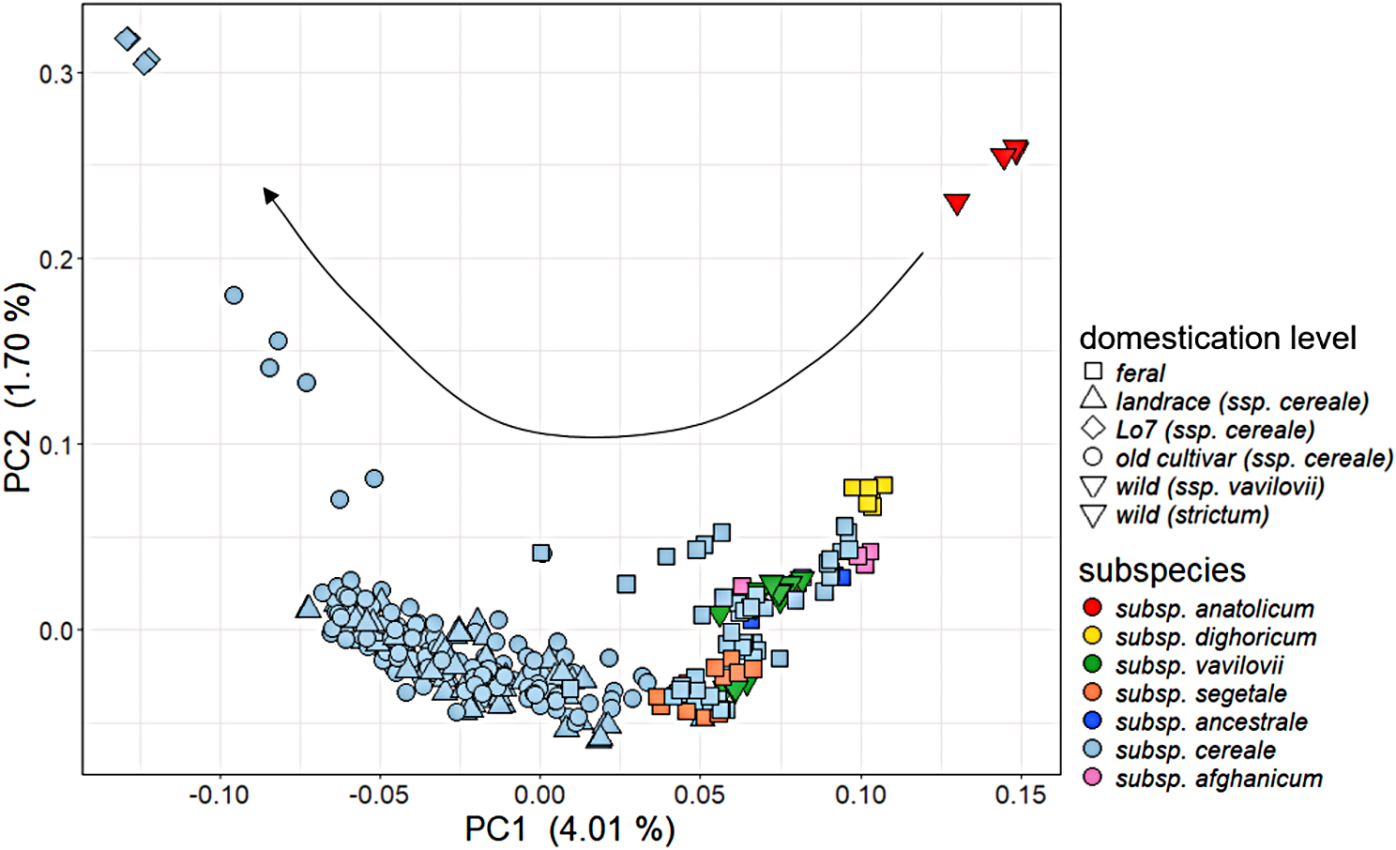
Gradient of divergence from wild to domesticated rye. PCA using 56,713 high-quality SNPs. Inbred lines, old cultivars, landraces, feral and wild rye are represented by distinct point shapes, while different rye subspecies are differentiated by various colours

In this study, we analysed a rye diversity panel comprising 64 rye genebank accessions of diverse geographic origins. The majority of accessions belonged to domesticated *S. cereale* subsp. *cereale*, including old cultivars and landraces (Supplementary Fig. 6A, Supplementary Table 1). The group of feral rye included various subspecies, while the group of wild rye was divided into three accessions of the wild progenitor *S. cereale* subsp. *vavilovii* and one accession of wild perennial *S. strictum* subsp. *anatolicum* [19, 25]. The population structure among 343 individuals was analysed based on principal component analysis (PCA) using 56,713 high-quality SNPs. We did not find distinctly separated genetic clusters in our population, with PC1 and PC2 explaining 5.71% of the total genotypic variation (Fig. 1). The lowest PC1 values correspond to an inbred line (Lo7), representing an outgroup among domesticated accessions, whereas the highest PC1 value corresponded to *S. strictum* subsp. *anatolicum* as the wild outgroup. Along PC1, old cultivars of domesticated *S. cereale* subsp. *cereale* clustered closest to the inbred line, followed by landraces (Fig. 1). In contrast, in descending order from the wild outgroup, a cluster of feral rye subspecies was detected, such as *dighoricum*, *afghanicum*, *ancestrale* and *segetale*, among also wild *S. cereale* subsp. *vavilovii* aligned. Interestingly, individuals of *S. cereale* subsp. *dighoricum* formed a cluster close to *S. strictum*, which both originating from Armenia, indicating cross-taxa admixture (Supplementary Fig. 6B; Supplementary Table 1), as already suggested by a previous study [47–49]. Apart from the Armenian material, *S. cereale* subsp. *afghanicum* originating from Afghanistan forms an exclusive cluster along PC4, whereas the other, mostly European accessions do not separate (Supplementary Fig. 6B). Our population showed a continuous gradient of divergence from wild to domesticated rye along PC1, without a clear separation among *S. cereale* between wild, feral and domesticated rye.

### Within-species variation of pollen and anther morphology

**Fig. 2:**
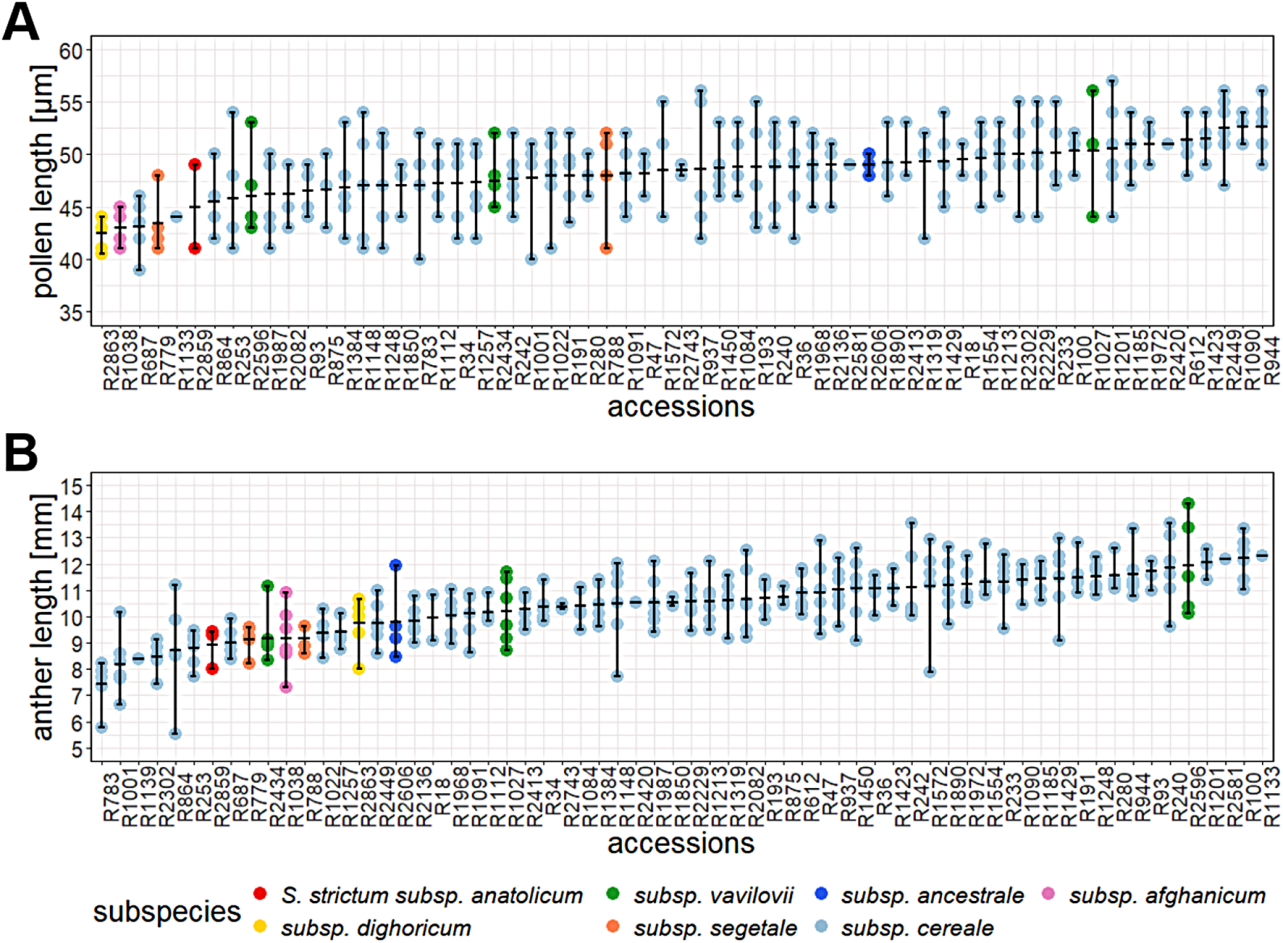
Intra-specific variation of pollen and anther morphology in rye. Distribution of pollen (**A**) and anther length values (**B**) according to rye accessions in ascending order, each dot represents the median value of an individual, highlighting variation between and among accessions. Different rye subspecies are distinguished by various colors

A major aim of our work was to explore the extent to which pollen and anther morphology vary within a species. To address this question, we analysed pollen and anther morphology across 298 and 328 individuals, respectively. Across a gradient of divergence from feral to domesticated rye, pollen length ranged between 39 and 57 μm (Fig. 2A) (Supplementary Table 5). As expected, the two tetraploid accessions R1090 and R944 displayed the highest pollen length, since it is known that the ploidy level is positively correlated with pollen size [48–50]. Pollen elongatedness, the ratio of pollen length and width, ranged between 1 and 1.3 indicating an ovoid, elongated shape of rye pollen, which is in agreement with previous reports [20, 26]. We found substantial within-species variation for pollen length and area, which are highly correlated (*r* = 0.98, *P* < 0.001) (Supplementary Table 9), with an average coefficient of variation of ~ 8% and ~ 15% respectively (Supplementary Table 5). This is extended by quantitative variations in pollen autofluorescence ranging from 28.1 to 31.4% (Supplementary Table 5). Pollen fluorescence intensities displayed strong positive correlations among each other (*r* = 0.84–1, *P* < 0.001) (Supplementary Table 9). Across our diversity panel, anther length ranged from 5.5 to 14.3 mm with a mean of 10.5 mm (Fig. 2B, Supplementary Table 5). Anther length showed modest positive correlation with pollen fluorescence and size traits (*r* = 0.14–0.32, *P* < 0.001). We measured pollen mass using quantitative phase imaging (QPI) in a subset of 27 individuals representing the range of pollen length values. Pollen mass ranged between 2.1 and 18.3 ng per grain (Supplementary Table 5), and had a strong positive correlation with pollen length (*r* = 0.89, *P* < 0.001) (Supplementary Table 9), demonstrating that larger pollen grains are also heavier. We modelled the distance across which pollen grains of a given size released from plants of a given height can be dispersed (Supplementary Fig. 7), which showed that subtle changes in pollen size (e.g., 40–55 μm) can have ecologically relevant effects on their distribution (e.g., 150–250 m).

### Impact of domestication on pollen and anther morphology

**Fig. 3:**
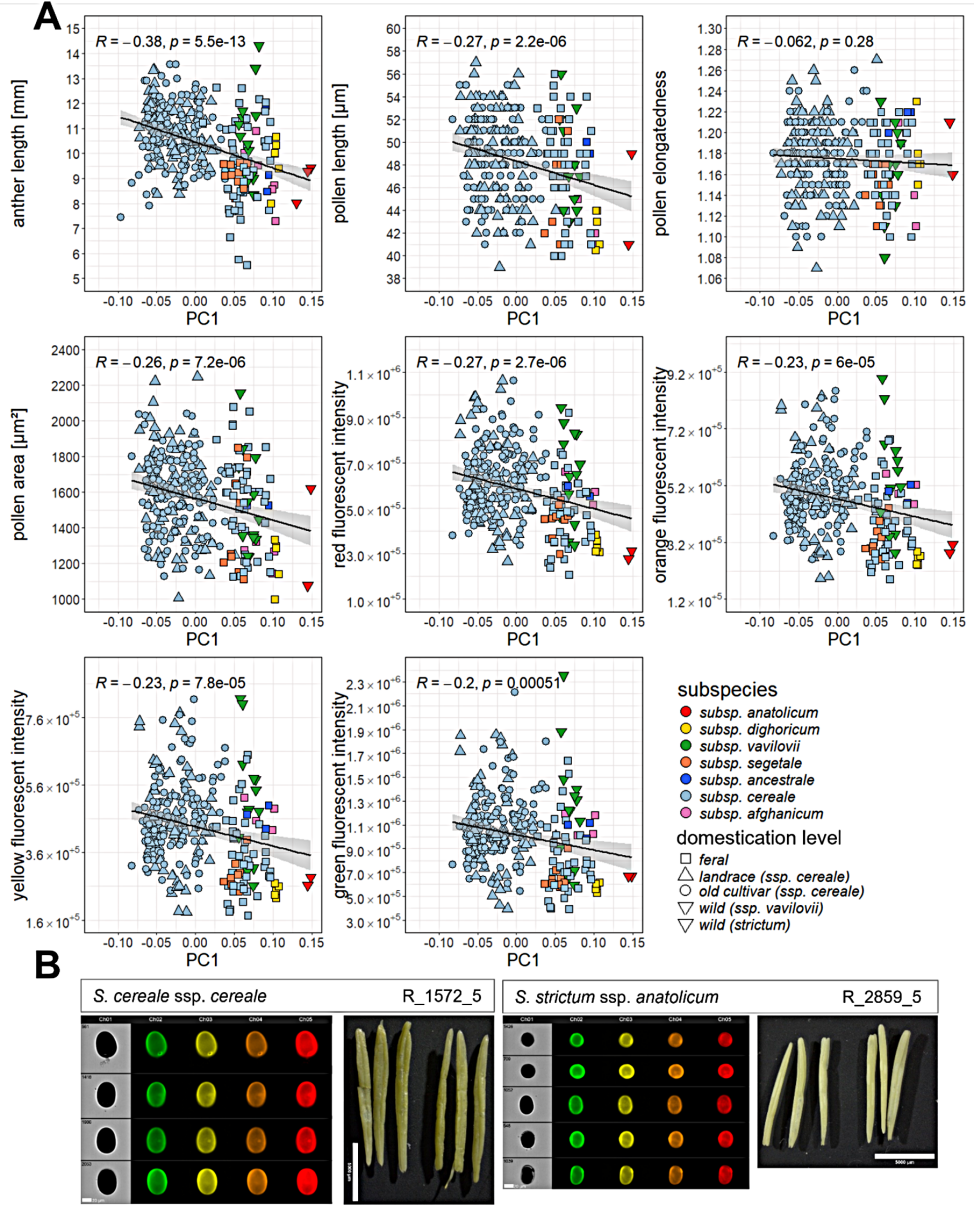
Pollen and anther length increase with the degree of domestication. (**A**) Correlation Scatterplots displaying the negative relationship between PC1 (response variable) and pollen and anther traits (explanatory variable), different colors and shapes indicating distinct subspecies and classifications into wild (*S. strictum*), feral, landrace and old cultivar, respectively. (**B**) Pollen images of brightfield, green, yellow, orange and red fluorescence channel (channel 1–5) from imaging flow cytometry (IFC) and images of anthers from microscopy for domesticated *S. c.* subsp. *cereale* and wild *S. s.* subsp. *anatolicum* with the respective rye individual designated and bars (20 μm for pollen, 5 mm for anthers) as size comparisons

Next, we analysed the relationship between the gradient of domestication along PC1 and pollen and anther morphology traits. We detected significant negative correlations between PC1 values and pollen and anther traits, the strongest negative with anther length (*r* = -0.38, *P* < 0.001) and the weakest negative with green fluorescence intensity (*r* = -0.2, *P* < 0.001) (Fig. 3A). Pollen elongatedness showed no significant correlation with PC1. This analysis revealed a tendency where a decrease in PC1 values, linked to a higher level of domestication, coincides with an increase in traits related to pollen and anther morphology (Fig. 3A and B). Motivated by that, we conducted pairwise comparisons between groups of domesticated (old cultivars and landraces), feral rye and the wild progenitor *S. c.* subsp. *vavilovii*, excluding wild *S. strictum* due to small group size (*n* < 4) (Supplementary Fig. 8). Old cultivars and landraces exhibited significantly higher (*P* < 0.05) trait values for anther length, pollen length, and fluorescence compared to feral rye. No significant differences were observed between domesticated and wild rye. Only for pollen fluorescences, we found significantly higher (*P* < 0.05) intensities for wild rye compared to feral. Pollen area and elongation showed no significant variation between groups. The groups of landraces, ferals and wild *S. c.* subsp. *vavilovii* further consisted of a mixture of spring and winter rye types, whereas old cultivars exclusively comprised winter rye (Supplementary Table 1). Again, for pollen elongatedness, we did not detect significant differences between spring and winter types (Supplementary Fig. 9). In the remaining pollen and anther traits, the winter types showed significant higher values compared to the spring types within landraces (*P* < 0.01) and within ferals (*P* < 0.05) for anther length and pollen fluorescences. Within wild rye only anther length was significantly increased (*P* < 0.05) in winter compared to spring types.

We further questioned whether differences in pollen fluorescence might result from local adaptation to UV radiation or heat stress and, thus, compared phenotypic values across centers of geographic origin (Supplementary Fig. 10). No significant differences were found within European samples, but we observed differences in fluorescence traits between Eastern European and Middle Eastern samples, and in orange fluorescence intensity between Southern European and Middle Eastern samples. However, these differences likely reflect domestication levels, as Middle Eastern rye lines include distinct feral rye subspecies, while European samples are primarily domesticated rye.

### Heritability and genome-wide association scans of pollen and anther morphology traits

We estimated the narrow-sense heritability (h^2^) of pollen and anther morphology traits, defined as the proportion of phenotypic variance explained by additive genetic effects. Among all traits measured, anther length showed the highest heritability (h^2^ = 0.81, SE = 0.08) and pollen elongatedness the lowest (h^2^ = 0.02, SE = 0.1). Pollen length and area displayed small heritabilities (h^2^ = 0.28–0.29, SE = 0.13–0.14). Pollen red, orange, yellow and green fluorescence intensities showed high heritabilities (h^2^ = 0.68–0.73, SE = 0.09–0.1).

**Fig. 4:**
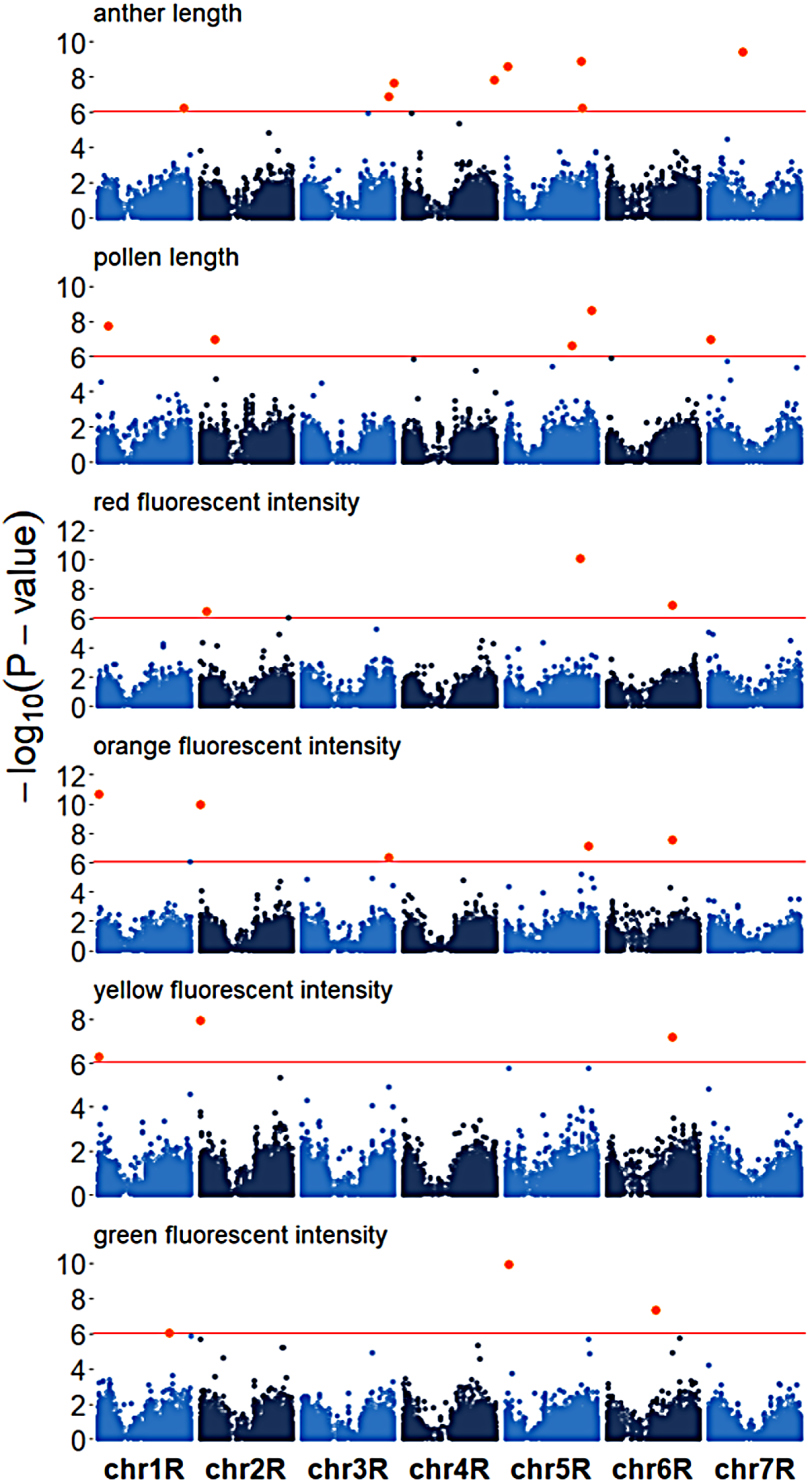
Genetic architecture of pollen and anther traits in rye. Manhattan plots of genome-wide association scans for anther length, pollen length, red, orange, yellow and green pollen fluorescence intensity. *P*-values are displayed on a–log10 scale and SNP markers above the Bonferroni-corrected significance threshold (red line) are highlighted in orange

Next we performed genome-wide association scans (GWAS) across all traits. We detected varying numbers of significant quantitative trait loci (QTL), ranging from three (e.g., pollen red fluorescence intensity) to eight (e.g., anther length) (Fig. 4). All QTL showed small effect sizes, with minor allele frequencies ranging between 5 and 48%, and explained phenotypic variances ranging between < 0.01–17% (Supplementary Table 10). To validate our detected QTL, we performed permutation tests for all traits by randomly assigning phenotypic information to genotypes. Thereby, only 6.3% of runs showed significant false-positive associations, which did not overlap with our detected QTL (Supplementary Table 3). The robustness of our GWAS was validated by repeated random subsampling (Supplementary Table 2), which demonstrated that the majority of QTL exhibited high detection rates expect for three QTL (QTL1_AL, QTL5_PL, QTL1_PGFI) with < 5% (Supplementary Table 10). While these could be considered potential false-positive associations, they did not overlap with the associations identified in the random permutation analysis. We also searched the remaining QTL for potential candidate genes within a genomic window of +/- 5 Mb around each significant SNP, which was based on the observed LD decay at 5 Mb inter-SNP distance in our population (Supplementary Fig. 5). Interestingly, two candidate genes for pollen and anther length, an O-acyltransferase (QTL3_PL, SECCE5Rv1G0341870) and an ABC-transporter protein (QTL8_AL, SECCE7Rv1G0483740) (Supplementary Table 4), respectively, were previously identified as domestication loci in rye, but without the assignment of a phenotypic trait [19, 25]. In conclusion, we detected various small-effect QTL suggesting a polygenic architecture of pollen and anther traits.

### Signatures of selection at pollen and anther morphology loci

**Fig. 5:**
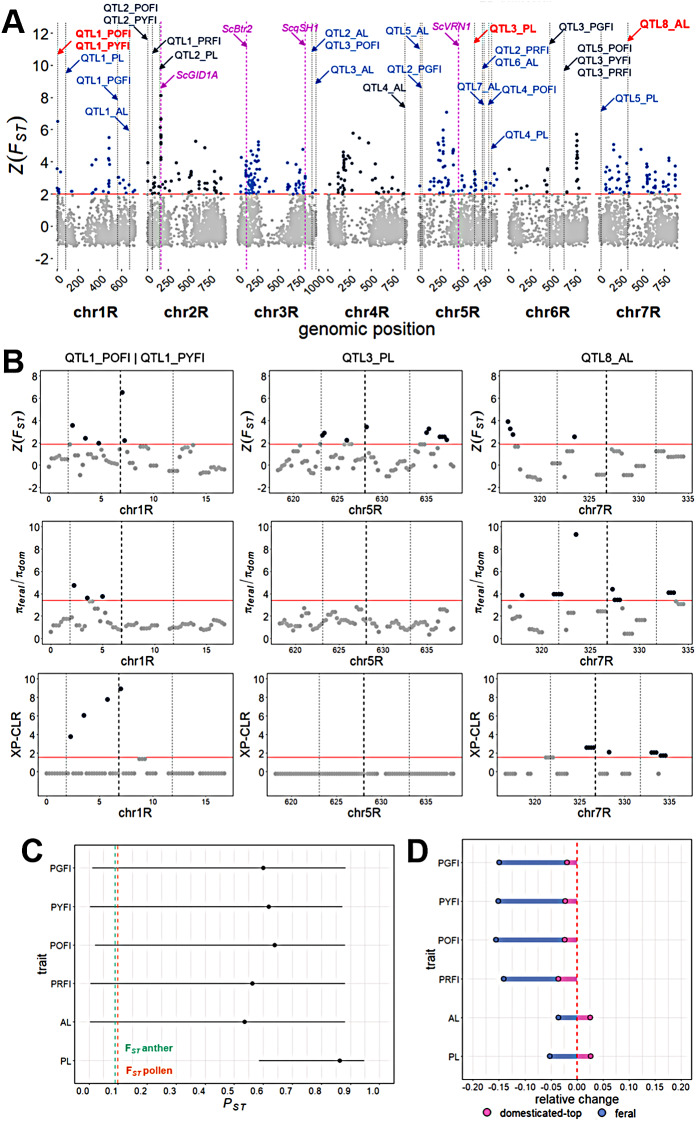
Pollen and anther traits were shaped by selection during the domestication of rye. (**A**) Distribution of the Z-transformed *F*_*ST*_ values between domesticated and feral outgroups along seven chromosomes of rye. The genome-wide threshold was defined by the top 5% region highlighted with a red horizontal line. Dark blue and grey dots highlight signals above and below the significance threshold, respectively. Grey dotted lines represent the genomic positions of the QTL, pink arrows indicate known orthologous domestication genes in rye. Blue and red arrows indicate the detected QTL. (**B**) Distribution of Z-transformed *F*_*ST*_, DRI *(π*_*feral*_*/π*_*domesticated*_*)* and XP-CLR values at three selected QTL in a +/- 5 Mb window around the SNP, highlighted by the two vertical grey dashed line. SNP position is indicated by the black vertical dashed line. The red horizontal line defines significance threshold of top 5% values, dark blue and grey dots highlight signals above and below the significance threshold, respectively. (**C**) *P*_*ST*_*-F*_*ST*_ scans of anther and pollen traits in feral and domesticated outgroups with available anther and pollen data. Bars indicate the lower- and upper-bounds, and dots indicate the mean *P*_*ST*_ of the 95% confidence intervals based on 1,000 bootstraps. Green (anther) and orange (pollen) vertical dashed lines indicate *F*_*ST*_ values calculated in groups with available phenotypic data. (**D**) Relative phenotypic changes for domesticated (pink) and feral (purple) outgroups in respect to the population mean (dashed red line)

Two of the genomic regions we found to be associated with pollen and anther length were previously described as domestication loci, without knowledge of the underlying phenotypic trait [19, 25]. This raised the question if our QTL detected for pollen and anther morphology were targeted during domestication and exhibited signatures of selection. To address this, we analysed chromosome-wide signatures of selection via Z(*F*_*ST*_), XP-CLR scores, and reduction of diversity index (*π*_*feral*_/*π*_*domesticated*_) using outgroups of domesticated and feral rye (Supplementary Table 6). As a control, we first tested if previously reported domestication loci were also detectable in our diversity panel. We observed significant Z(*F*_*ST*_) values at four previously identified domestication genes in rye, barley, rice and wheat (*ScGID1A*, *ScBtr2*, *ScqSH1*, *ScVRN1*) [19, 51–54] (Fig. 5A). Considering our QTL, we detected significant Z(*F*_*ST*_) values at three QTL for anther length, one QTL for pollen length, and two QTL each for red, orange, and yellow pollen fluorescence intensity (Supplementary Table 7). At two QTL for anther length, as well as orange and yellow pollen fluorescence intensity, signatures of selection were detected by all three metrics. These loci comprise the candidate ABC-transporter protein associated with anther length on chromosome 7R, and an ankyrin repeat protein (SECCE1Rv1G0001960) associated with orange and yellow pollen fluorescence on chromosome 1R, respectively (Fig. 5B). Our previously detected domestication locus harbouring an O-acyltransferase associated with pollen length only showed a significant signal by Z(*F*_*ST*_). Next, we performed *P*_*ST*_ -*F*_*ST*_-analysis within the domesticated and feral outgroups with available phenotypic data (Supplementary Table 8). For all traits, we detected *P*_*ST*_ > > *F*_*ST*_ (Fig. 5C), indicating that the observed phenotypic variations between domesticated and feral rye (Fig. 5D) were shaped by selection during domestication rather than genetic drift. In conclusion, we could detect signatures of selection for anther and pollen trait QTLs throughout rye domestication.

## Discussion

In this study, we have provided compelling evidence that within-species variation in pollen and anther morphology were shaped by domestication in rye (*Secale cereale* L.). We showed that domesticated rye displays longer anthers and larger pollen compared to feral rye. We detected several small-effect QTLs for pollen and anther traits with a subset of these loci overlapping with previously identified domestication loci, for which the underlying traits were unknown [19, 25]. A *P*_*ST*_-*F*_*ST*_ analysis suggested that pollen and anther traits were under selection throughout rye domestication. Population genomic analyses revealed signatures of selection at 37% of all identified loci. Here, we discuss in more detail the within-species variation and polygenic architecture of pollen and anther morphology in rye as well as the reasons these traits were selected upon during domestication.

### Gradient of domestication in *Secale cereale*

Consistent with previous findings, we did not identify distinct genetic clusters separating domesticated and feral rye within *Secale cereale*. Instead, we observed a gradient of genetic divergence corresponding to the degree of domestication [18, 19, 23, 25]. This gradient indicates that old cultivars and landraces are closely aligned with inbred lines like Lo7, which represents contemporary breeding material, while feral rye of various subspecies and wild *S. c. subsp. vavilovii* exhibit genetic proximity to the wild perennial *S. strictum*. The lack of distinct clusters indicates strong gene flow between ferals and domesticates due to the outcrossing and self-incompatible nature of rye. We did not examine spindle brittleness of our material, a key trait differentiating between domesticated and wild lines. However, our 64 rye accessions were part of a larger diversity panel analysed in recent studies, which investigated spindle brittleness, confirming the wild-growing status of *S. c. subsp. vavilovii* and *S. strictum* and characterising ferals [54, 55].

### Pollen and anther morphology traits correlate with gradient of domestication

In palynological studies, occurrence of cereal pollen was used as an indicator for agricultural land use. These studies demonstrated that pollen of domesticated cereals was larger compared to those of wild grasses [55, 56]. Here, we expand on these observations by demonstrating intra-specific pollen size variation (CV = 8%) in rye with an increase in pollen length from feral to domesticated accessions (Fig. 3A). Especially old cultivars and landraces showed significantly higher pollen size compared to feral rye (Supplementary Fig. 8). We showed that pollen size and mass are positively correlated (*r* = 0.89, *P* < 0.001; Supplementary Table 9). For domesticated rye, this would mean an increased pollen mass compared to pollen of feral rye, eventually affecting pollen dispersal by wind [7]. Our modeling approach demonstrated that even subtle changes in pollen size can have ecologically relevant effects on their distribution distance (Supplementary Fig. 7). For wild, scattered rye populations, it might be beneficial to have smaller pollen overcoming farther distances for successful fertilisation. Cultivated rye populations would have been grown at higher planting densities, eliminating the need for pollen being dispersed across great distances. However, plant height is an additional factor affecting pollen dispersal distance [7]. Cultivated rye tends to have higher plant height than weedy rye [19], which might compensate the shorter dispersal distance due to higher pollen size. Pollen size also correlates with the amount of stored nutrients, meaning a decreased nutrient content due to smaller size [12–14]. A higher nutrient level could affect pollen germination by accelerating pollen tube growth [26, 56–61] in domesticated rye.

Anther length was shown to positively correlate with pollen number in several species [26, 57–62]. A higher pollen output increases the chances of successful fertilisation, suggesting that anther length plays a role in efficient pollen dispersal alongside pollen size. We found substantial within-species variation (CV = 13%) for anther length, whereby domesticated rye accessions, like old cultivars and landraces, displayed significantly higher anther length (*P* < 0.001) compared to feral rye (Supplementary Fig. 8). In our work, anther length was positively correlated with pollen length (*r* = 0.32, *P* < 0.001) and pollen mass (*r* = 0.4, *P* < 0.05) (Supplementary Table 9), but these rather modest correlations also suggest an independent genetic architecture of those traits. In this context, the likely influence of genotype-environment interactions on pollen and anther traits cannot be ruled out but was not evaluated in this study, as all rye genotypes were grown in a single environment.

### Potential ecological impact of pollen fluorescence intensities

Besides size parameters, we were able to investigate pollen fluorescence intensities using multi-spectral imaging flow cytometry. In general, pollen autofluorescence is known to vary between species and can be attributed to protection against UV-radiation [34, 63–65]. Previous studies demonstrated autofluorescence of sporopollenin, a major component of the pollen exine, in the 400–650 nm range, which is triggered by UV-absorbing phenylpropanoid derivates [66–69]. This wavelength range spectrum would best correspond to the measured green fluorescence emission intensities (528/65 nm) excited at 488 nm, where we also detected the highest overall pollen fluorescence intensity (Fig. 3A, Supplementary Table 5). Besides sporopollenin, various components of the intine and exine, such as proteins, flavonoids, phenolic compounds, and carotenoids display autofluorescence [69, 70]. Pollen red fluorescence intensity (PRFI) (702/85 nm) and pollen orange fluorescence intensity (POFI) (610/30 nm) can be attributed to alkaloids, with autofluorescence in the orange (585–620 nm) and red (625–700 nm) range [70]. Pollen yellow fluorescence intensity (PYFI) (577/35 nm) might be associated with carotenoids displaying autofluorescence within 500–560 nm and flavonoids like quercetin (500–585 nm) [70]. However, the variations in fluorescence excitation spectra across different studies limits the comparability of fluorescence emission spectra. Fluorescence intensities and spectra can also be affected by chemical treatment of pollen, influencing the composition of the pollen wall [68]. In *Ericaceae*, pollen fluorescence intensity increased and shifted to longer wavelength in acetolysis washing solution whereas samples washed in alcohol and distilled water produced similar spectra [71]. In our case, fluorescence emission could have been affected by storage in ethanol: acetic acid (3:1), but since all samples experienced the same treatment, at least within-species fluorescence intensity variation is not expected to be influenced.

Our observed within-species variation in pollen fluorescence intensities suggest differences in the chemical composition of pollen and the pollen exine, serving as a protective layer against e.g., UV-radiation and enabling local adaptations. However, we only observed significant differences in pollen fluorescences between European and Middle Eastern accessions, likely stemming from differences in level of domestication, but not among European lines (Supplementary Fig. 10). To fully understand the relationship between pollen fluorescence intensities and the chemical composition of pollen and the exine, pollen surface metabolome analyses will be required.

#### Genetic architecture of pollen and anther morphology traits

We observed moderate heritabilities for pollen length (h^2^ = 0.29) and high heritabilities for anther length (h^2^ = 0.81), consistent with previous studies [72–74]. Interestingly, substantial heritable genetic variation for pollen size was reported for some species of cultivated plants (*Brassica rapa*, *Phaseolus vulgaris* and *Raphanus sativus*), but absent in a number of wild species (*Spergularia marina*, *Mimulus guttatus* and *Campanula rapunculoides*) [75–77]. This indicates an influence of domestication on additive genetic variation of pollen size [73, 74]. Our *P*_*ST*_-*F*_*ST*_-analysis within outgroups of domesticated and feral rye confirmed that phenotypic variations of pollen and anther length were shaped by selection during domestication and not by genetic drift (Fig. 5C).

We were able to detect various small-effect QTL for pollen and anther morphology, with explained phenotypic variances ranging between < 0.01–17% (Supplementary Table 10), emphasizing the polygenic architecture of those traits. These findings are consistent with previous studies in faba bean (*Vicia faba* L.), where two QTL related to pollen size explained phenotypic variances between 11.5 and 17.6% [78].Similarly, in wheat (*Triticum aestivum*), a QTL for anther length accounted for 19–21% of the observed phenotypic variance (Song et al., 2018). In *Arabidopsis thaliana*, on the other hand, a single QTL associated with pollen number variation explained ~ 20% of the observed phenotypic variance [79]. In our work, a QTL on chromosome 4R related to anther length was of special interest since it showed the second-highest explained variance of 14% and the highest detection rate of 91% (Supplementary Table 10). Past studies showed that the long arm of chromosome 4R harbours QTL associated with anther length [61, 80, 81], even increasing anther size in wheat by 16% when introgressing rye chromosome 4R [61]. Furthermore, a restorer of fertility locus (Rfp1), which confers increased anther size in rye, was previously mapped to the long arm of chromosome 4R at approximately 880–890 Mb (Lo7 assembly) [21, 82–84], which is in proximity to an anther length QTL localized at ~ 887 Mb we detected in our work. We found an acetate kinase (SECCE4Rv1G0293930) as a candidate gene with minimal distance to this QTL (Supplementary Table 4), but previously proposed candidate genes for Rfp1 are mitochondrial transcription termination factors (mTERF) and pentatricopeptide repeat (PPR) RNA-binding factors [85], which are also detected +/- 1 Mb around this QTL. However, orthologues of the acetate kinase are highly expressed in anthers and pollen in wheat (TraesCS6A02G015100, TraesCS6B02G021700, TraesCS6D02G017900) [86–88]. Further on, two candidate genes for pollen and anther length, an O-acyltransferase (QTL3_PL, SECCE5Rv1G0341870) and an ABC-transporter protein (QTL8_AL, SECCE7Rv1G0483740), respectively, were previously identified as domestication loci in rye, but without the assignment of a phenotypic trait [19, 25]. Wheat orthologues of the ABC-transporter (TraesCS7A02G268800, TraesCS7B02G167100, TraesCS7D02G269400), are highly expressed in anthers, whereas orthologues for the O-acyltransferase (TraesCS5A02G322100, TraesCS1B02G402900, TraesCS5D02G328600) are enriched in spikelets during booting and ear emergence stage [86–88]. We detected selection signatures at the two QTL loci for the ABC transporter and O-acyltransferase, as well as at 37% of all QTL identified, suggesting that pollen and anther traits have been shaped throughout rye domestication.

## Conclusions

Taken together, we present novel domestication phenotypes in rye, including anther and pollen length, as well as pollen fluorescence. We were able to detect multiple small effect loci for these traits, of which 37% showed signatures of selection. Our results show that during the domestication of rye, selection for longer anthers with larger pollen occurred. Longer anthers would have the advantage of a higher pollen production per anther [26, 57–62], and therefore a higher pollen output increasing the chances of successful fertilization, and with that, seed set [6]. Larger pollen would have more nutrients stored, which could accelerate pollen tube growth during pollen germination [12–14]. Increased pollen output and faster fertilization would enhance the likelihood of successful fertilization, thereby reducing the risk of ergot infection, making anther and pollen length potential domestication traits in rye. Our study extends our knowledge of the genetic architecture underlying quantitative variations in pollen and anther morphology and further unravels the domestication history of rye.

## Electronic supplementary material

Below is the link to the electronic supplementary material.

Supplementary Material 1

Supplementary Material 2

## Data Availability

Data of this study are available at the European Nucleotide Archive under accession number PRJEB75239 (https://www.ebi.ac.uk/ena/browser/view/PRJEB75239).
