# Supplementary material for: Pollen and anther morphological variation in rye was shaped by domestication
Source: BMC Plant Biol. 2025 Mar 27;25:389. doi: 10.1186/s12870-025-06416-x (PMC11948849; doi:10.1186/s12870-025-06416-x)
Supplement: Supplementary file 1 — Supplementary Material 1 [file 12870_2025_6416_MOESM1_ESM.pptx]

## Slide 1
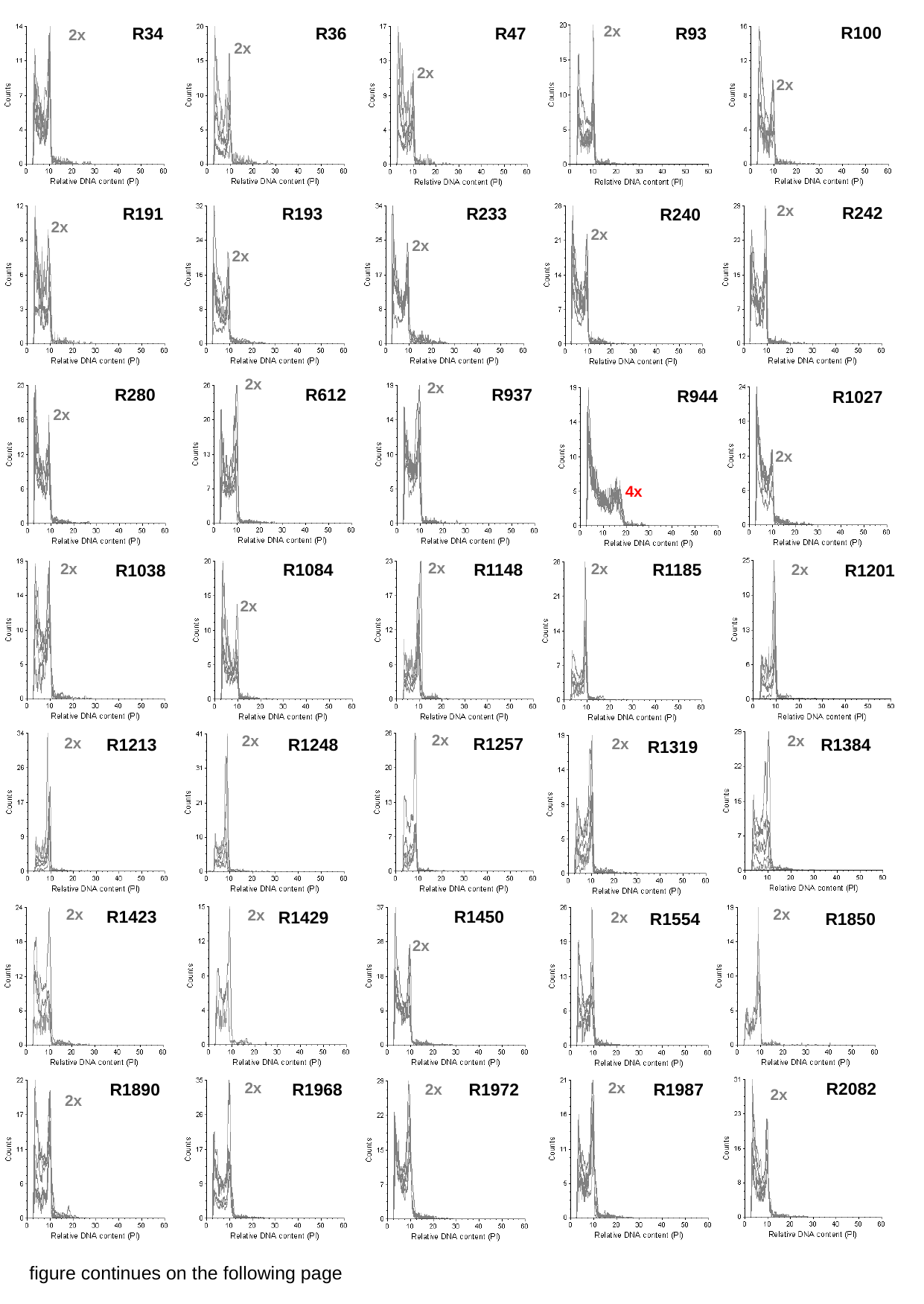

2x
R100
R34
R93
R36
R47
2x
2x
2x
2x
2x
R242
R193
R191
R233
R240
2x
2x
2x
2x
2x
2x
R937
R612
R280
R944
R1027
2x
2x
4x
2x
2x
R1148
R1185
2x
R1084
R1038
R1201
2x
2x
2x
2x
2x
R1257
2x
R1213
R1248
R1384
2x
R1319
2x
2x
2x
R1450
R1423
R1429
2x
R1554
R1850
2x
2x
2x
R2082
R1968
R1987
R1890
R1972
2x
2x
2x
figure continues on the following page

## Slide 2
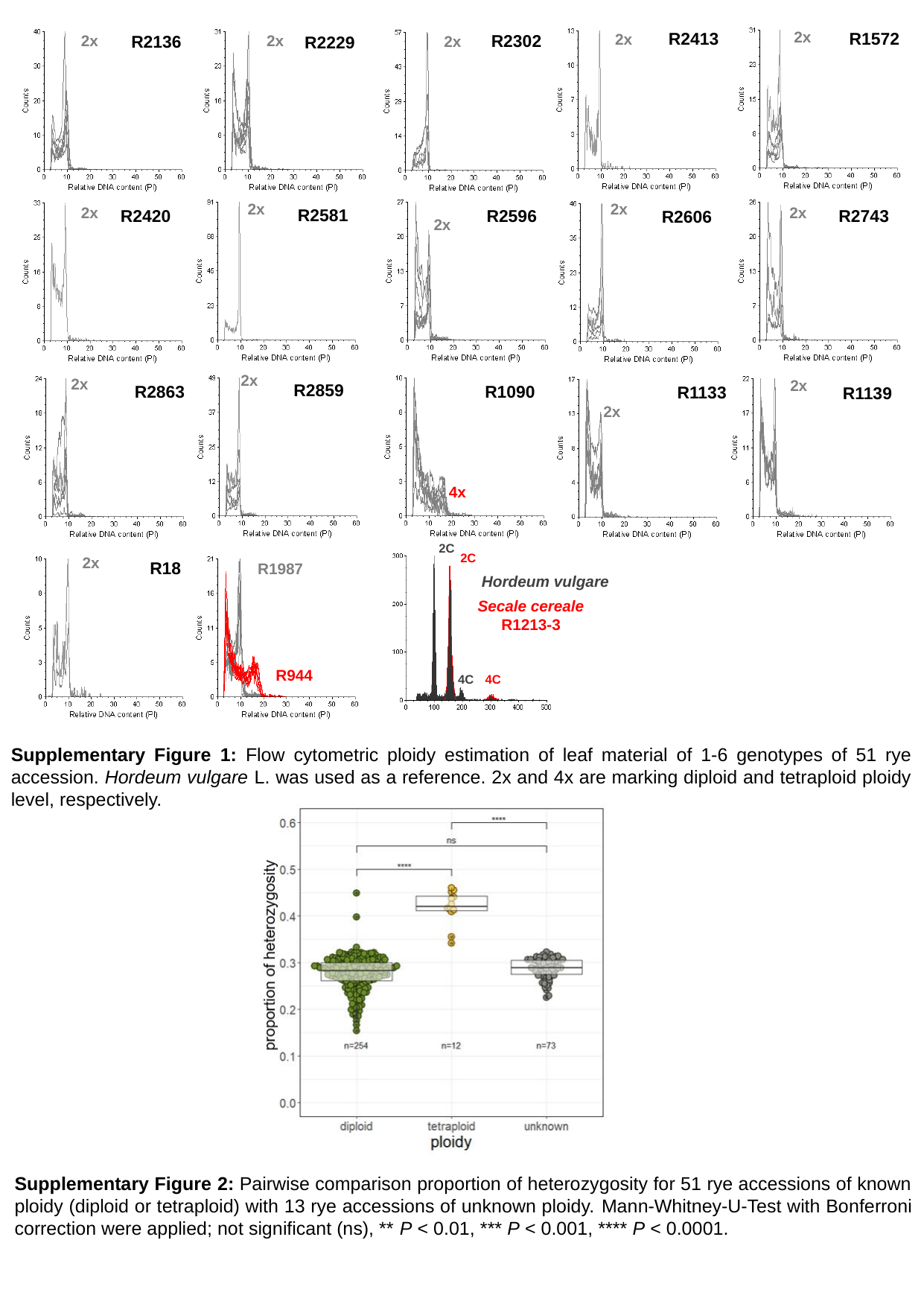

2x
R2413
R1572
2x
R2302
R2136
2x
2x
R2229
2x
2x
2x
2x
2x
R2581
R2743
R2596
R2420
R2606
2x
2x
2x
2x
R2859
R2863
R1090
R1133
R1139
2x
4x
2C
2C
2x
R18
R1987
Hordeum vulgare
Secale cereale
R1213-3
R944
4C
4C
Supplementary Figure 1: Flow cytometric ploidy estimation of leaf material of 1-6 genotypes of 51 rye accession. Hordeum vulgare L. was used as a reference. 2x and 4x are marking diploid and tetraploid ploidy level, respectively.
Supplementary Figure 2: Pairwise comparison proportion of heterozygosity for 51 rye accessions of known ploidy (diploid or tetraploid) with 13 rye accessions of unknown ploidy. Mann-Whitney-U-Test with Bonferroni correction were applied; not significant (ns), ** P < 0.01, *** P < 0.001, **** P < 0.0001.

## Slide 3
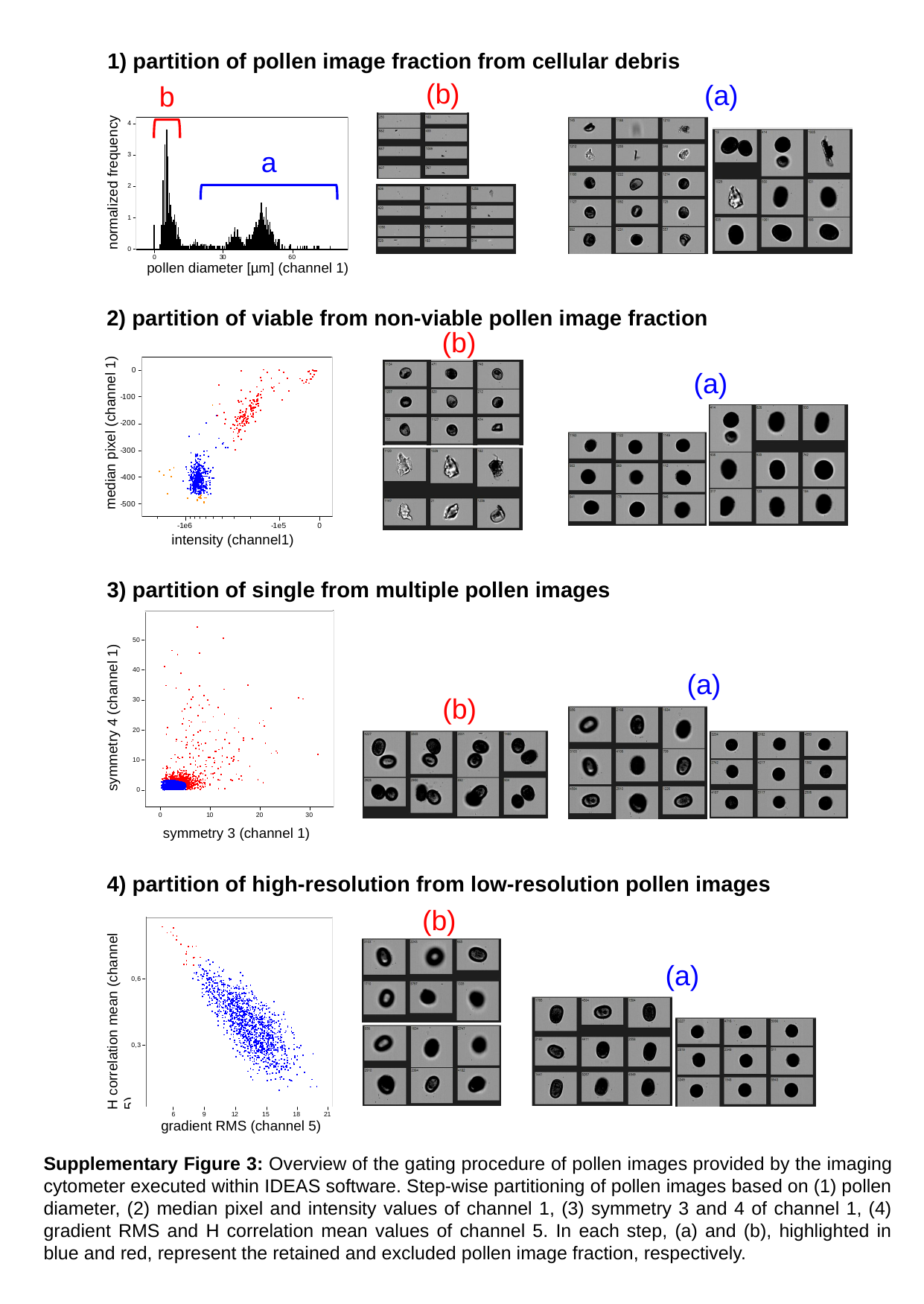

1) partition of pollen image fraction from cellular debris
(b)
(a)
b
a
normalized frequency
pollen diameter [µm] (channel 1)
2) partition of viable from non-viable pollen image fraction
(b)
(a)
median pixel (channel 1)
intensity (channel1)
3) partition of single from multiple pollen images
(a)
(b)
symmetry 4 (channel 1)
symmetry 3 (channel 1)
4) partition of high-resolution from low-resolution pollen images
(b)
(a)
H correlation mean (channel 5)
gradient RMS (channel 5)
Supplementary Figure 3: Overview of the gating procedure of pollen images provided by the imaging cytometer executed within IDEAS software. Step-wise partitioning of pollen images based on (1) pollen diameter, (2) median pixel and intensity values of channel 1, (3) symmetry 3 and 4 of channel 1, (4) gradient RMS and H correlation mean values of channel 5. In each step, (a) and (b), highlighted in blue and red, represent the retained and excluded pollen image fraction, respectively.

## Slide 4
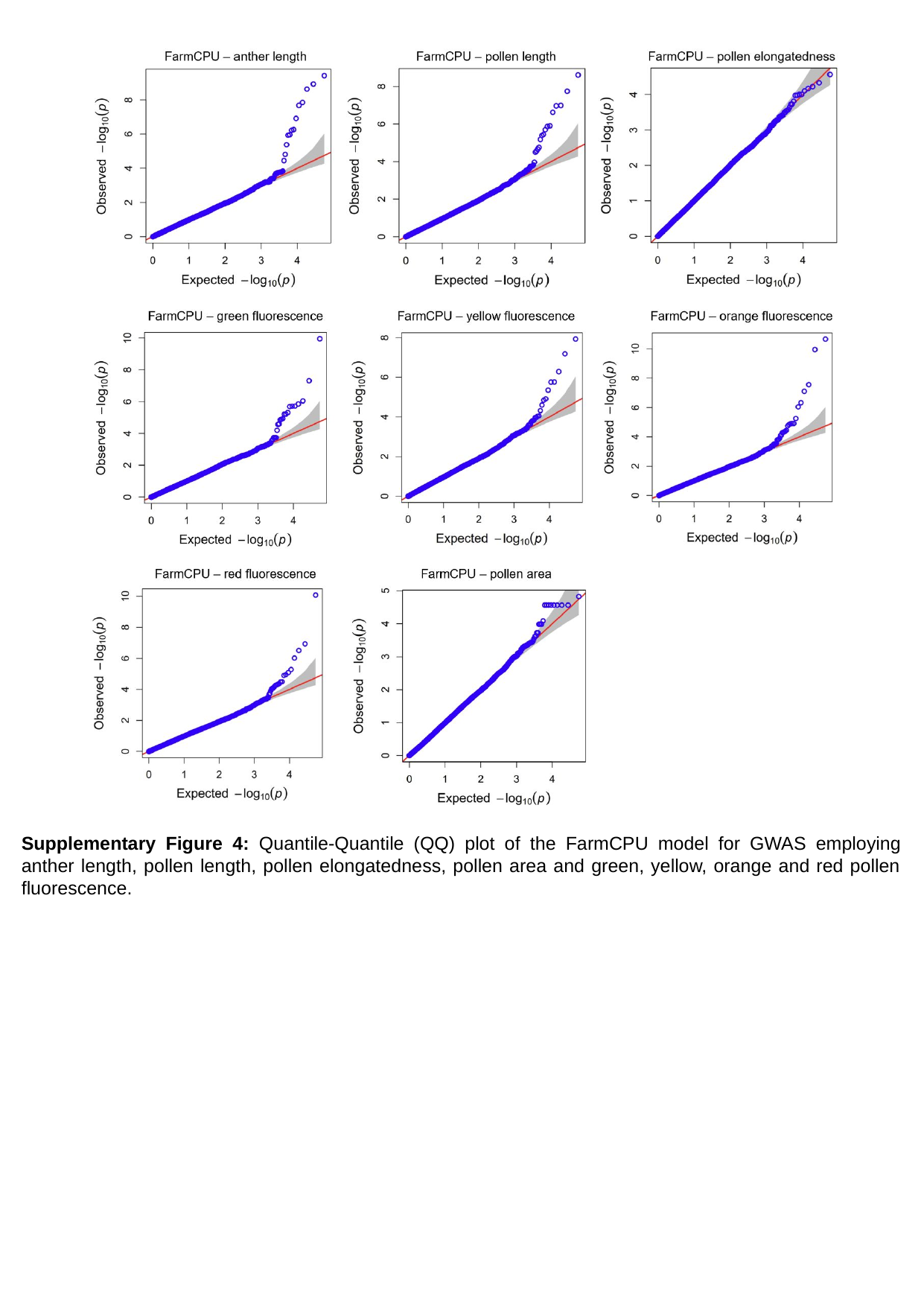

Supplementary Figure 4: Quantile-Quantile (QQ) plot of the FarmCPU model for GWAS employing anther length, pollen length, pollen elongatedness, pollen area and green, yellow, orange and red pollen fluorescence.

## Slide 5
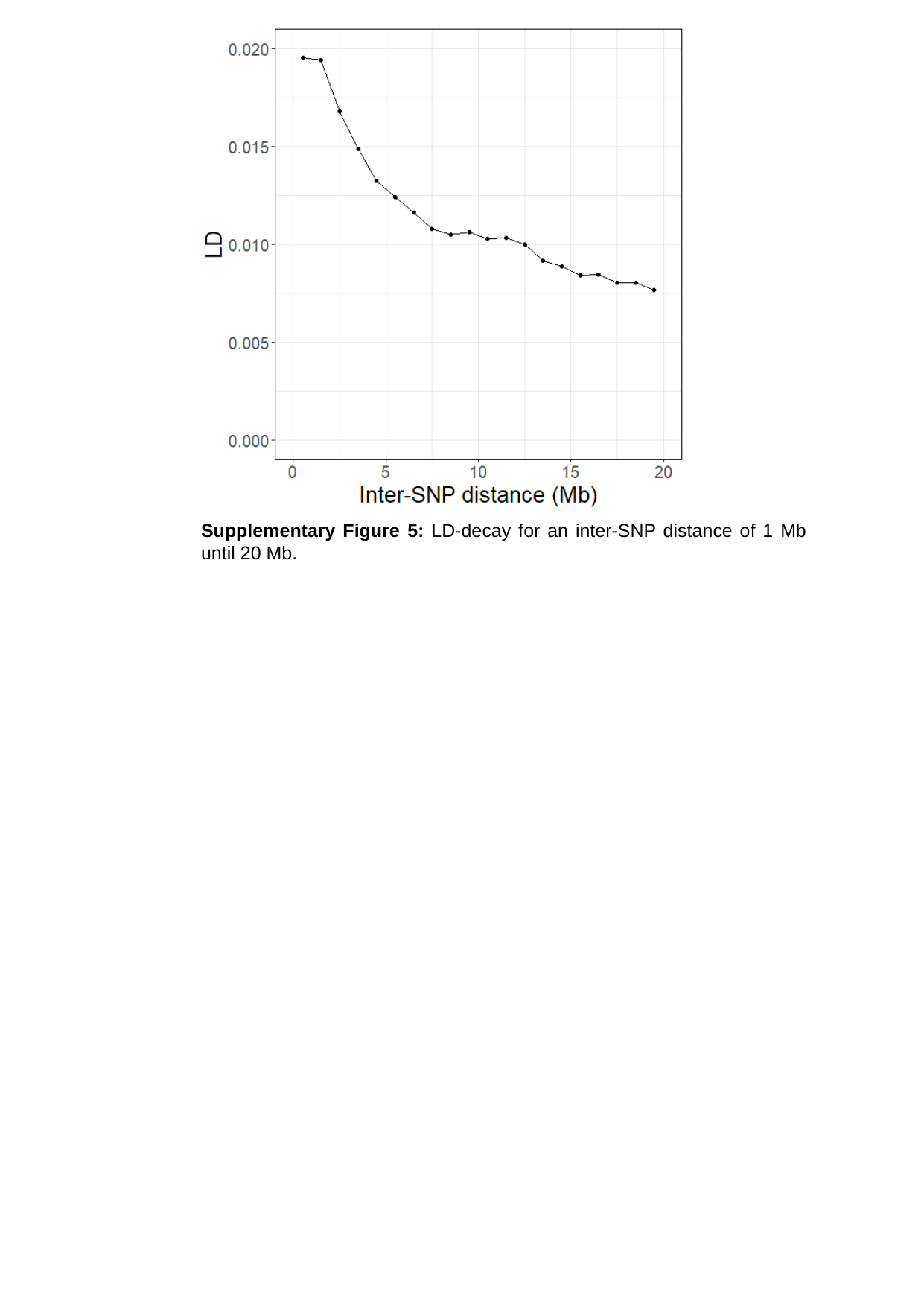

Supplementary Figure 5: LD-decay for an inter-SNP distance of 1 Mb until 20 Mb.

## Slide 6
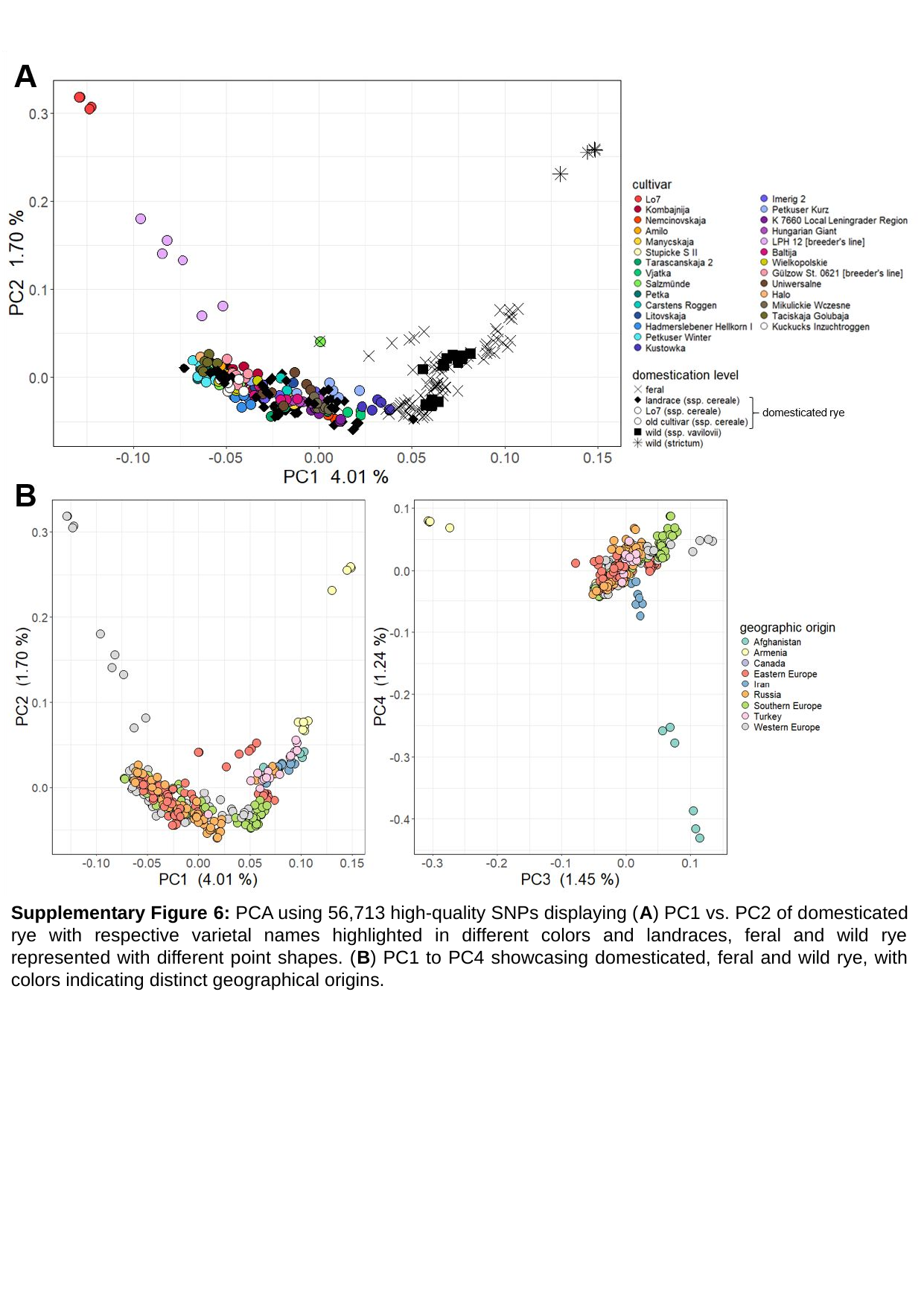

Supplementary Figure 6: PCA using 56,713 high-quality SNPs displaying (A) PC1 vs. PC2 of domesticated rye with respective varietal names highlighted in different colors and landraces, feral and wild rye represented with different point shapes. (B) PC1 to PC4 showcasing domesticated, feral and wild rye, with colors indicating distinct geographical origins.

## Slide 7
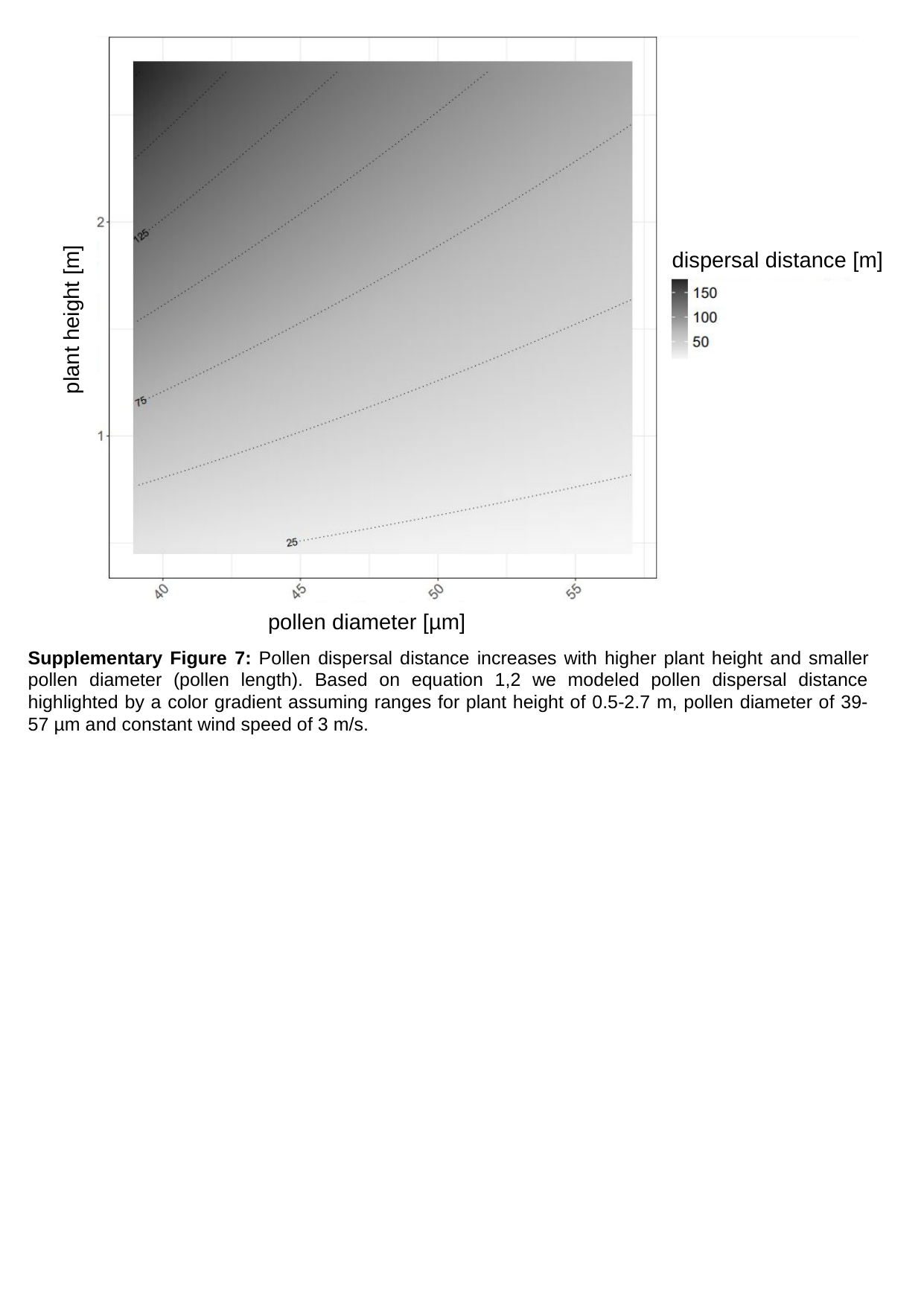

dispersal distance [m]
plant height [m]
pollen diameter [µm]
Supplementary Figure 7: Pollen dispersal distance increases with higher plant height and smaller pollen diameter (pollen length). Based on equation 1,2 we modeled pollen dispersal distance highlighted by a color gradient assuming ranges for plant height of 0.5-2.7 m, pollen diameter of 39-57 µm and constant wind speed of 3 m/s.

## Slide 8
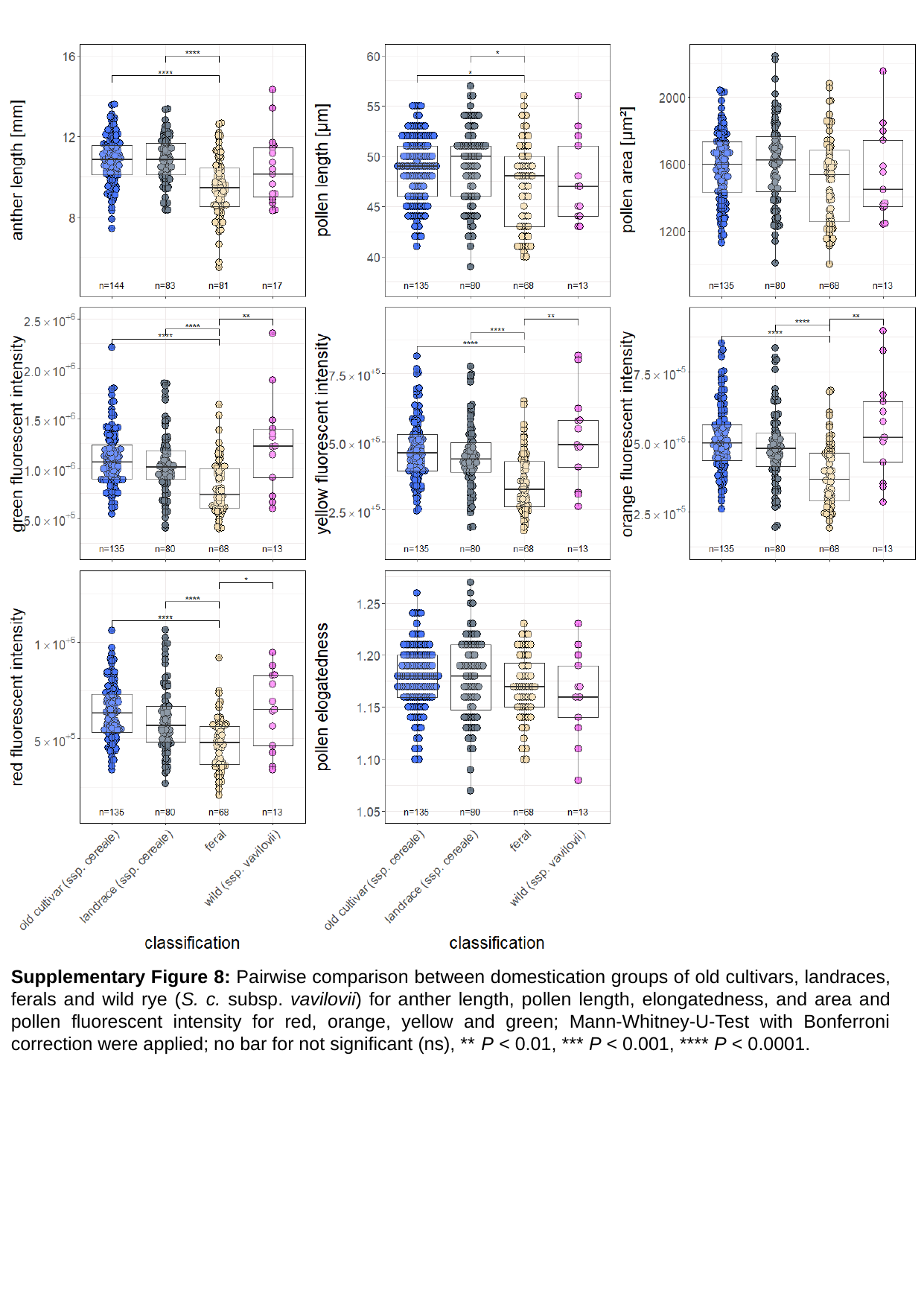

Supplementary Figure 8: Pairwise comparison between domestication groups of old cultivars, landraces, ferals and wild rye (S. c. subsp. vavilovii) for anther length, pollen length, elongatedness, and area and pollen fluorescent intensity for red, orange, yellow and green; Mann-Whitney-U-Test with Bonferroni correction were applied; no bar for not significant (ns), ** P < 0.01, *** P < 0.001, **** P < 0.0001.

## Slide 9
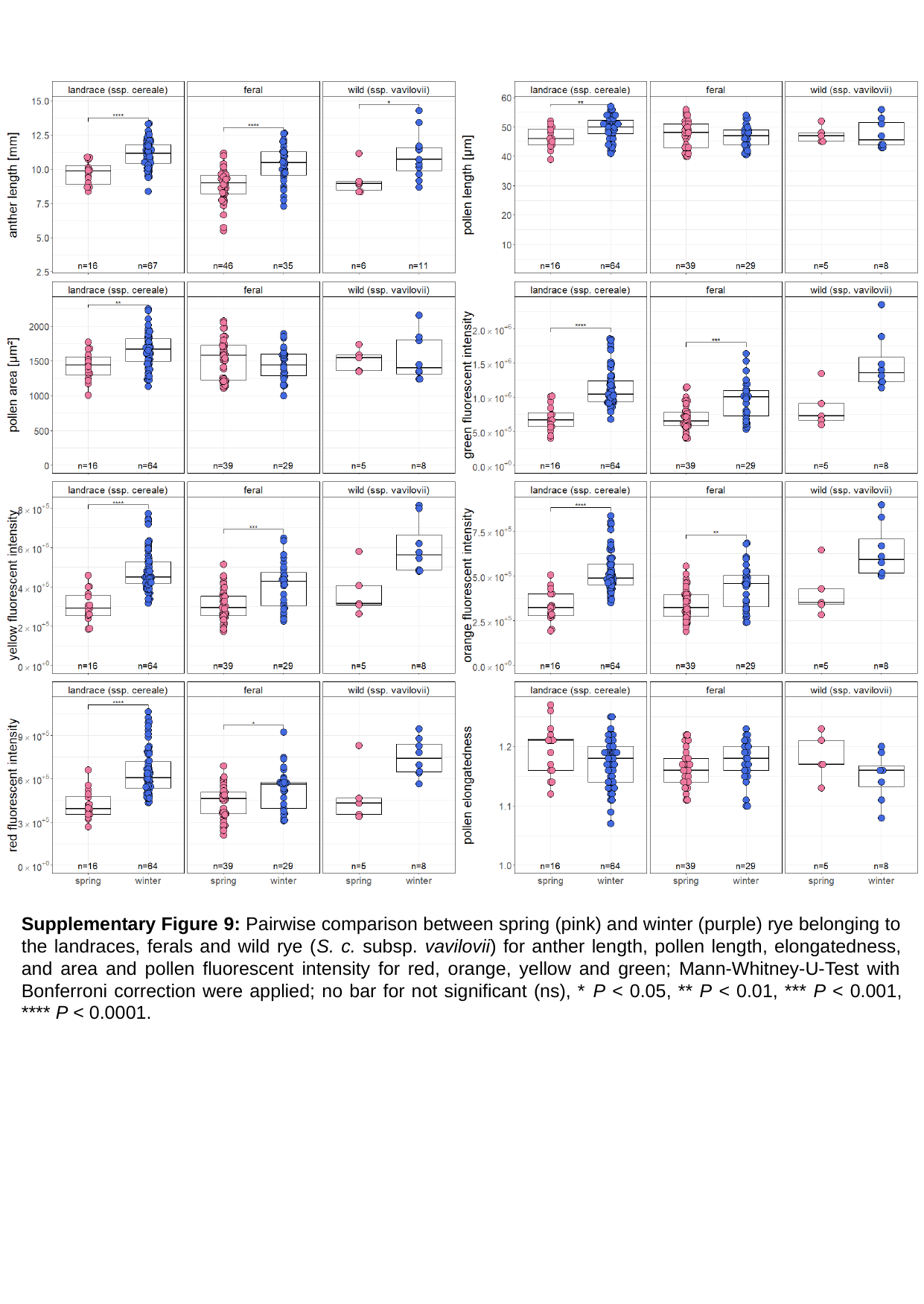

Supplementary Figure 9: Pairwise comparison between spring (pink) and winter (purple) rye belonging to the landraces, ferals and wild rye (S. c. subsp. vavilovii) for anther length, pollen length, elongatedness, and area and pollen fluorescent intensity for red, orange, yellow and green; Mann-Whitney-U-Test with Bonferroni correction were applied; no bar for not significant (ns), * P < 0.05, ** P < 0.01, *** P < 0.001, **** P < 0.0001.

## Slide 10
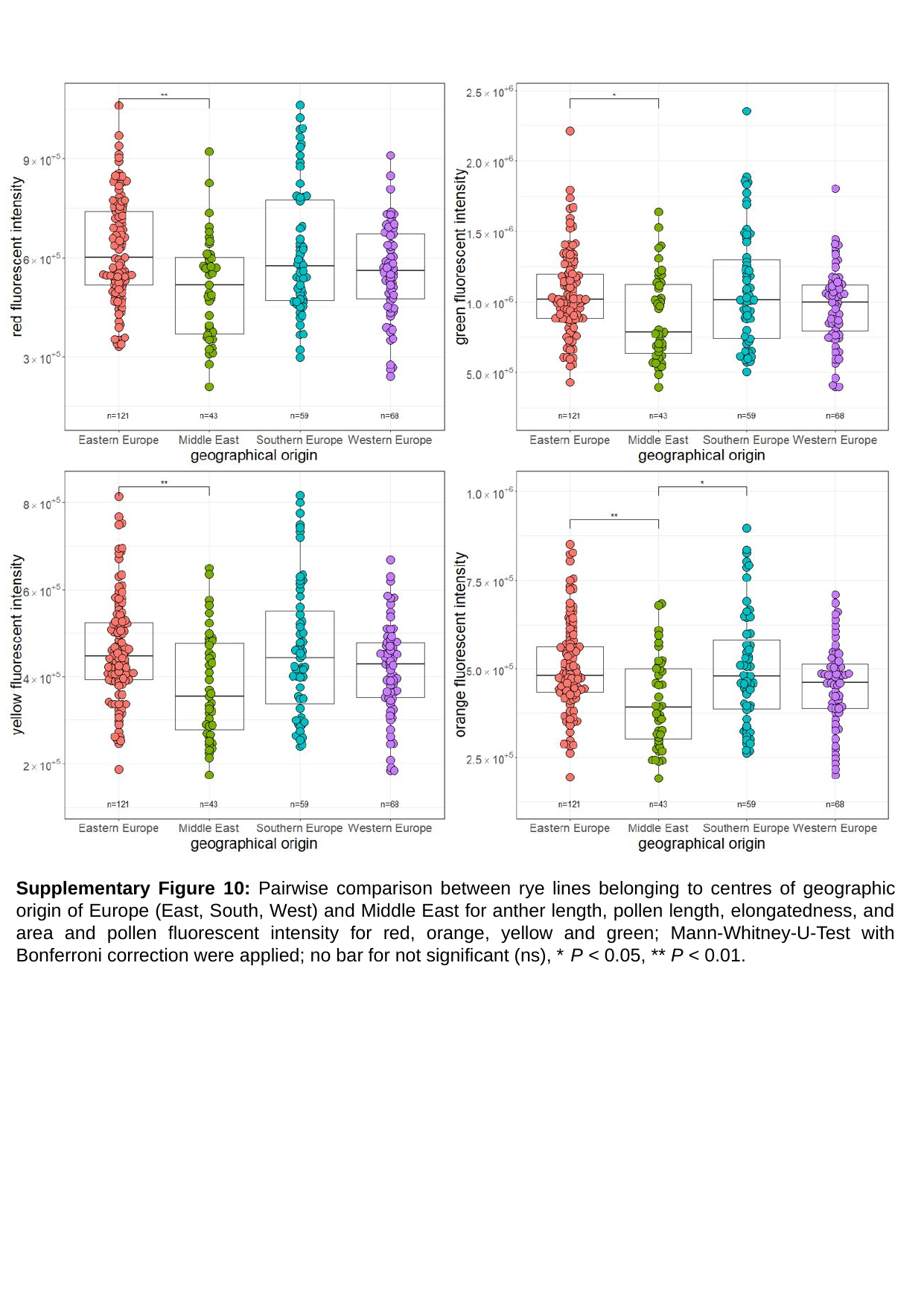

Supplementary Figure 10: Pairwise comparison between rye lines belonging to centres of geographic origin of Europe (East, South, West) and Middle East for anther length, pollen length, elongatedness, and area and pollen fluorescent intensity for red, orange, yellow and green; Mann-Whitney-U-Test with Bonferroni correction were applied; no bar for not significant (ns), * P < 0.05, ** P < 0.01.
